# Comparison of Antibacterial Activity of* Lactobacillus plantarum* Strains Isolated from Two Different Kinds of Regional Cheeses from Poland: Oscypek and Korycinski Cheese

**DOI:** 10.1155/2017/6820369

**Published:** 2017-05-24

**Authors:** Aleksandra Ołdak, Dorota Zielińska, Anna Rzepkowska, Danuta Kołożyn-Krajewska

**Affiliations:** Department of Food Gastronomy and Food Hygiene, Warsaw University of Life Sciences-SGGW, Warsaw, Poland

## Abstract

Oscypek and korycinski are traditional Polish cheeses, exclusively produced in Tatra and in Podlasie region, respectively, produced from raw, unpasteurized milk. The 29* Lactobacillus plantarum* strains were isolated on MRS agar from 12 cheese samples and used as a material for study. The main purpose of the work was to assess the antimicrobial properties and recognition of selected strains for the unique antagonistic activity and preservation role in food. It has been found that the highest antimicrobial activity was observed in the case of* L. monocytogenes *strains; however, the level of that activity was different depending on the* Lb. plantarum* strain. Strains from oscypek produced broad spectrum, and a few strains isolated from korycinski cheese produced a narrow spectrum of antimicrobial compounds, other than organic acids and hydrogen peroxide. Moreover, the antagonistic activity shown by* Lb. plantarum* strains is connected with the source from which a given strain was isolated. Strains isolated from oscypek cheese represented stronger activity against* L. monocytogenes*, whereas strains isolated from korycinski cheese were more active against* E. coli*. Strains* Lb. plantarum* Os13 and Kor14 could be considered as good candidates for protective cultures to extend durability of food products.

## 1. Introduction

Growth in consumer interest in natural and low-processed food causes the necessity to explore the new ways of food preservation; for example, the use of natural components in an innovative manner is observed in recent years [1]. Lactic acid bacteria, due to metabolic processes, produce different antimicrobial compounds such as organic acids (primarily lactic and acetic acid), hydrogen peroxide, and antimicrobial peptides, including bacteriocins [2, 3]. Some* Lactobacillus* spp. show strong antagonistic activity against spoilage and pathogenic bacteria and fungi [1]. As indicated by references, antimicrobial action of LAB is mainly connected with the organic acids production and lowering of pH of environment [2–5]. However, it was also shown that ability of bacteriocin or bacteriocin-like substances synthesis has positive impact on antagonistic activity [4, 5].* Lactobacillus* spp. are one of the most common types of lactic acid bacteria in food and feed product. Technological properties and health benefits of numerous strains from this group make them promising starter and probiotic cultures [1, 6, 7]. Due to its technological skills and also valuable probiotic features* Lactobacillus plantarum* is one of the most miscellaneous species in* Lactobacillus* genus. Furthermore, it was shown that many* Lb. plantarum* strains have the ability to provide unique metabolic processes like the vitamins synthesis or production of host immunomodulatory particles [6, 8].* Lb. plantarum* is also a versatile and heterogeneous species that is observed in different environmental niches, including dairy products, meat, and vegetables. This mesophilic group of lactobacilli may be the dominant species in several types of cheese [9, 10].

Cheese is one of the oldest known kind of processed food. Many regions of the world have traditional cheeses, from various types of milk, with or without flavor additives such as spices and also with or without salt. Consumer preference of cheese made from raw milk is growing, due to its more intense flavor than pasteurized milk cheeses. Anyhow, the dominant group of raw milk cheese is LAB, and there were foodborne illnesses outbreaks linked to this type of food. These cases resulted in a decrease of consumer trust to cheeses prepared from raw milk. However, it has been widely described that raw milk cheeses are microbiologically hazardous because of lack of thermal inactivation of pathogenic microbiota. Nevertheless, Brooks et al. [11] showed that all raw milk cheeses were negative for pathogens such as* Listeria monocytogenes*,* Salmonella*, and* Escherichia coli* O157:H7. Moreover, exactly pasteurized milk cheeses were source of infection of a high number of* Listeria*-associated foodborne illnesses outbreaks in Germany from 2006 till 2007 [11].

The aim of this work was to assess the antimicrobial properties of* Lactobacillus plantarum* strains isolated from two different types of regional cheeses produced in different region of Poland and recognition of these strains for the unique antagonistic activity and preservation role in food.

## 2. Material and Methods

### 2.1. Characterization of Selected Regional Cheeses

Oscypek is traditional Polish cheese, exclusively produced in Tatra region. The unpasteurized and salted sheep's milk is turned into curd, which is rinsed many times with boiling water and squeezed. The addition of raw cow's milk is possible at the beginning of process: two kinds of milk are mixed together before salting. This process is precisely described by the protected recipe. After that, the bulk is pressed into spindle-shaped, wooden decorative forms. The forms are placed in a barrel filled in brine for 1-2 days. After that, the forms are placed in dry special wooden hut. Cheeses can be smoke for up to 14 days at temperature above 40°C.

Korycinski cheese is the local kind of rennet cheese produced from the raw cow's whole milk in Korycin, in the Podlasie region. Production process was unchanged for years: raw milk is heated and treated with salt and rennet. When the whey is separated, the cheese is formed and rubbed with salt. After that the brine is dripped off and the cheese is placed onto dry shelf for the maturation process.

### 2.2. Isolation and Identification of LAB Cultures

Six samples of traditional oscypek cheese and six samples of korycinski cheese, obtained from 3 different parts of production, were collected. Each time ten grams of cheese samples was transferred aseptically into 90 mL Ringer's solution and homogenized thoroughly. Serial dilutions were made and spread-plated on De Man, Rogosa, and Sharpe, MRS agar (Merck, Darmstadt, Germany). The typical colonies on the MRS agar plates were isolated and phenotypically identified according to Gram stain assay and catalase test.

Isolates identified initially as Gram-positive lactobacilli were transferred to genetic identification using PCR method and 16S rDNA sequencing. Genomic DNA of the LAB strains was extracted using the reagent kit Genomic AX Bacteria Mini Spin (A&A Biotechnology, Poland) according to producer's manual. The primers couple 27F/1492R (5′-AGA GTT TGA TCC TGG CTC AG-3′/5′- GGT TAC CTT GTT ACG ACT T-3′) was used for the amplification of 16S rDNA. After amplification, PCR products were examined formerly for the expected size on 1% agarose gel and visualized using ethidium bromide under UV light. 16S rDNA sequencing was executed with the Illumina technique. The BLAST program was used for sequence comparison. The strains classified to the genus* Lactobacillus* and species* Lb. plantarum* have been subjected to further testing.

### 2.3. Bacterial Growth Conditions

Bacterial cultures were stored at −80°C with the 20% glycerol (w/v).* Lb. plantarum* cultures were cultivated at 37°C for 24 h in MRS broth (Lab M, United Kingdom) under semiaerobic conditions and tested for antagonistic activity against indicator strains according to Sip et al. [5] and Hartmann et al. [12].

Indicator strains used for assessment of antimicrobial activity were three* Listeria monocytogenes* strains (ATCC 7644, ATCC 19111, and ATCC 15313), two species from* Enterobacteriaceae* family (*Salmonella enteritidis* ATCC 13076,* Escherichia coli *ATCC 10536), and also* Bacillus subtilis* and* Enterococcus faecium* strains (food isolates). Cultures were carried to solid selective medium: Palcam Agar (Lab M, United Kingdom) for* Listeria*, TBX Agar (Merck, Darmstadt, Germany) for* E. coli*, and BGA (Oxoid, United Kingdom) for* Salmonella*. The frozen cultures of* B. subtilis* and* E. faecium* were transferred onto Nutrient Agar (Lab M, United Kingdom). After incubation single colonies of each indicator strain were transferred to nutrient broth (Biokar Diagnostic, Noack, Poland) and cultivated from 24 to 48 h at 37°C under aerobic condition.

### 2.4. Antimicrobial Properties of* Lactobacillus plantarum* Strains

#### 2.4.1. Well Diffusion Method

The* Lb. plantarum* strains were described for antagonistic activity using the well diffusion method. Antimicrobial effectiveness of whole bacteria culture (WBC), cell-free supernatant (CFS), and catalase treated cell-free neutralized (CFN) supernatant was examined. The* Lb. plantarum* 299v was used as a reference strain.

The cultures were centrifuged (6000 ×g, 10 min) and filter sterilized (0.2 *μ*m) and the antimicrobial properties of these cell-free supernatants were tested to measure only the influence of extracellular metabolites of LAB. After that the supernatants were treated with catalase (300 IU/mL, Sigma Aldrich, Poland) and neutralized with 1 M NaOH (to eliminate the influence of organic acids, low pH, and hydrogen peroxide). These CFN (cell-free neutralized) supernatants were examined in further tests. CFN supernatants which exhibited antagonistic activity to indicator strains were assessed as potentially synthesizing bacteriocins or bacteriocin-like substances.

Sterile Petri dishes were poured with Nutrient Agar (Lab M, United Kingdom) and inoculated with 200 *μ*L culture of each indicator strain severally (concentration 6 log CFU/mL). After that wells of 5.5 mm diameter were cut out and filled with whole cultures, supernatants, and also CFN supernatants. Plates were incubated at 37°C for 48 h and the diameters of inhibition growth zones were measured. Antimicrobial activity (*x*) was calculated as follows:* x *=* D* −* d*, where* D *is the inhibition zone diameter and* d *is the well diameter. Antimicrobial activity (*x*) was characterized and classified based on the inhibition growth zone diameters and described as slight (*x* < 4 mm diameter), medium (*x* = 4–8 mm), high (*x* = 8–12 mm), and very high (*x* > 12 mm).

#### 2.4.2. Coculture in Skim Milk

Seeing that the tested* Lb. plantarum* strains have been isolated from dairy products, it was decided to examine their antimicrobial properties in the milk matrix. The study included one strain from each source. The Os13 and Kor14 strains were selected, based on high antimicrobial activity obtained in well diffusion assay. As an indicator strains* L. monocytogenes* ATCC 19111,* S. enteritidis* ATCC 13076, and* E. coli* ATCC 10536 were used.

Ten mL of skim milk (Mlekovita, Poland) was inoculated with 100 *μ*L of dilution in Buffered Peptone Water (Oxoid, United Kingdom) indicator strain culture, to obtain ~100 bacteria cells. Then 100 *μ*L of* Lb. plantarum* (Os13 or Kor14) whole culture has been added. The initial count of each bacteria strain was checked by a plate count technique. Samples inoculated only with the indicator strains were also prepared as controls. Samples were incubated at 37°C for 16 h, 24 h, and 64 h. In each mentioned time point samples dilutions were plated onto solid medium: Palcam for count of* L. monocytogenes*, BGA for count of* Salmonella*, and TBX for* E. coli*. Count of* Lactobacillus* was examined on MRS agar. The plates were incubated for 24–48 hours in 37°C. After incubation, typical colonies on each solid medium were counted and results were transformed to logarithmic scale.

### 2.5. Statistical Analysis

Statistical analysis was performed using STATISTICA15 (StatSoft Inc.).

## 3. Results and Discussion

### 3.1. Isolation and Identification of LAB Cultures

The 87 different strains of bacteria were isolated on MRS agar from 12 cheese samples. The 29 strains identified as* Lactobacillus plantarum *were selected for further research, including 17 strains isolated from oscypek and 12 strains isolated from korycinski cheese. All strains selected for the study displayed 98–100% 16S rDNA sequence similarity to* Lb. plantarum* and were classified as belonging to this species. Sequences are available under NCBI number KY744437-KY744453 for* Lb. plantarum* Os strains and KY744455-KY744466 for* Lb. plantarum* Kor strains.

*Lb. plantarum* is the most numerous species among nonstarter lactic acid bacteria identified in ripened cheeses [13]. Many studies demonstrate that the number of* Lb. plantarum* cells, relatively small at the beginning of the production process (in comparison with other species), increases considerably during cheese ripening [9, 13]. Moreover, it was found that* Lb. plantarum*, along with other species, that is,* Lb. brevis, Lb. parabuchneri, Lb. helveticus, Lb. paracasei, Lb. fermentum*, and* Lb. pentosus*, is a typical component of the microflora of Slovenská bryndza [13].

### 3.2. Antimicrobial Properties of* Lactobacillus plantarum* Strains

#### 3.2.1. Well Diffusion Method

**Figure 1:**
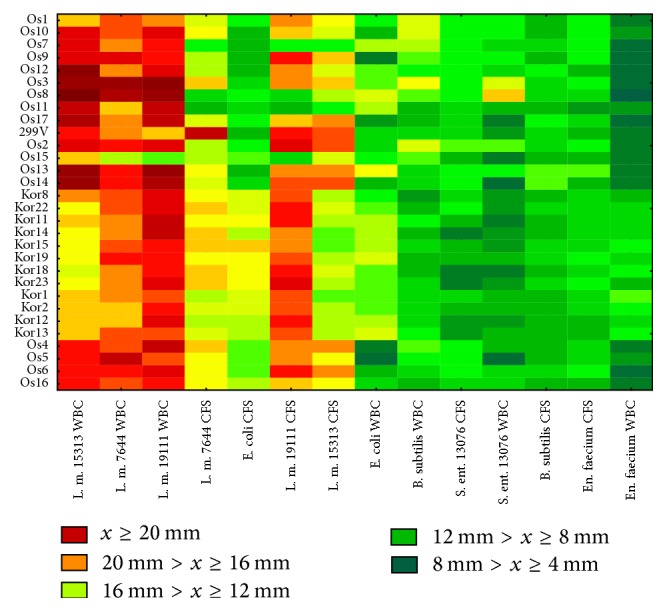
Inhibition zones map including all tested* Lb. plantarum *strains and all indicators strains. Explanation: L.m. 15313:* Listeria monocytogenes* ATCC 15313; L.m. 7644:* Listeria monocytogenes* ATCC 7644; L.m. 19111:* Listeria monocytogenes* ATCC 19111; E. coli:* Escherichia coli* ATCC 10536; B. subtilis:* Bacillus subtilis*; En. faecium:* Enterococcus faecium*; S. ent. 13076:* Salmonella enteritidis* ATCC 13076; form of* Lb. plantarum* strain used: WBC: whole bacteria culture; CFS: cell-free supernatant;* x*: antimicrobial activity.

The diffusion method applied allowed an initial assessment of the antimicrobial potential of the strains studied. The strains were characterized by very high, high, and moderate antagonistic activity towards the indicator microorganisms used in the study, which is illustrated in Figure 1 and in the Supplementary Material (see Supplementary Material available online at https://doi.org/10.1155/2017/6820369). The highest antimicrobial activity was observed in the case of* L. monocytogenes *strains; however, the level of that activity was statistically significantly different depending on the* Lb. plantarum* strain and the* L. monocytogenes *strain used in the study. The largest zones of inhibition of culture (WBC) growth by* Lb. plantarum* were observed on average for* L. monocytogenes* ATCC 19111 (18.27 mm ± 2.70 mm) and the smallest ones for* En. faecium* (6.71 mm ± 2.20 mm). The three times lower antagonistic activity towards* En. faecium *observed in this study may result from a higher tolerance of this species to pH and the presence of organic acids.* En. faecium* is a lactic acid bacteria species and is also frequently identified, especially in fresh cheese [14].

The very high anti-*Listeria* activity of lactic acid bacteria has already been demonstrated by many authors [5, 12, 15, 16]. The research of Sip et al. [5] revealed that strains isolated from golka, a regional Tatra cheese obtained from cow's milk, were characterized by very high antagonistic activity towards* L. monocytogenes*, and the observed diameters of zones of inhibition were similar to those recorded in this experiment [5].

The research of Li et al. [17] revealed that the CFS of* Lb. plantarum* LZ206 showed anti-*Listeria* activity similar to that observed in this study, where the zones of inhibition were 15–20 mm in diameter. Antagonistic activity towards* B. subtilis* was also recorded at a similar level (10–15 mm). However, the authors [17] found that the supernatant of* Lb. plantarum* LZ206 was more than twice as active against a* Salmonella enterica *strain as the* Lb. plantarum* strains were active against* S. enteritidis *in this study, which may be related to the presence of fragments coding plantaricin, a broad-spectrum bacteriocin, in the genome of* Lb. plantarum* LZ206. On the other hand, the study of Hartmann et al. [12] did not demonstrate any inhibitory effect of* Lb. plantarum* on the growth of* S. enterica*.

In our study, statistically significant differences were revealed in the antagonistic activity displayed by the cultures (WBC) and the supernatants (CFS) of the* Lb. plantarum* strains examined (Figure 1). The WBCs usually showed a higher antagonistic activity than the CFSs, especially towards three* L. monocytogenes *strains. This may suggest that this antagonistic activity depends on a higher degree on the presence of live* Lactobacillus plantarum *cells capable of metabolic processes, probably including the synthesis of active antimicrobial substances. The research of Sip et al. [5] revealed no tendency for antagonistic activity to decrease as bacteria cells were removed; in the case of some strains, no antagonistic activity was noted then (zone of inhibition diameter: 0 mm), and in the case of other strains, the activity was even higher (the zone of inhibition diameter for the CFSs was 3–6 mm larger than for the whole cultures) [5].

In the case of indicator strains other than those of the* L. monocytogenes* species, the observed differences between the levels of antagonistic activity of the cultures (WBCs) and the supernatants (CFS) were not so wide and the intensity of this activity was comparable for both forms.

In our study, a very low antagonistic activity of catalase treated cell-free neutralized (CFN) supernatant was indicated, which on the one hand suggests that the strains studied may be capable of synthesizing bacteriocins or bacteriocin-like inhibitory substances (BLIS) and on the other hand confirms the demonstrated, also by other authors, most significant influence of decreased culture pH on the antimicrobial properties of* Lactobacillus* strains [1, 18]. The neutralization and catalase treatment of the supernatant resulted in its antagonistic activity being reduced on average more than 10 times. In the study of Arena et al. [1], the inhibitory activity of the supernatant was observed to slightly decrease after the supernatant was heated to 80°C and treated with catalase. The neutralization of the supernatant, in turn, resulted in the loss of inhibitory activity towards the model strains [1].

**Figure 2:**
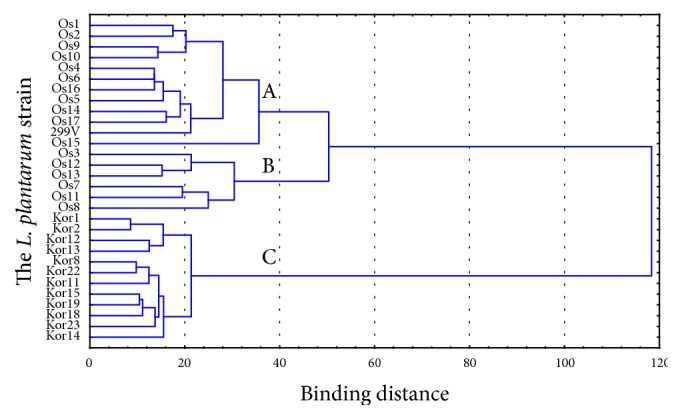
Dendrogram generated for clusters using Ward method, Euclidean distances; A, B, C: groups considered as statistically different (*p* < 0.05) by ANOVA.

**Figure 3:**
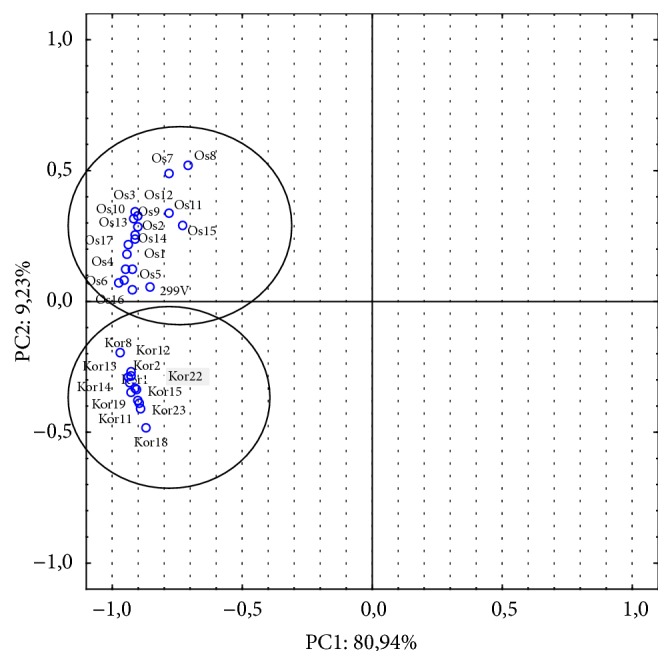
Principal component analysis (PCA) based on antimicrobial activity of 29 of* L. plantarum *strains (WBC and CFS). Two first components explained 90.17% of the total variance: PC1 explained 80.94% and PC2 explained 9.23% of the total variance, respectively.

**Figure 4:**
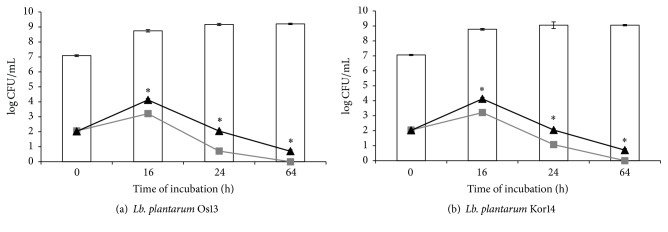
Number of the viable counts [log CFU/mL] of* L. monocytogenes* and* Lb. plantarum* Os13 and Kor14 in cocultures as follows: 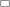: count of* Lb. plantarum* Os13 (a) and Kor14 (b); 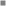:* L. monocytogenes* in coculture with* Lb. plantarum* Os13 (a) and Kor14 (b); 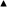:* L. monocytogenes* in control sample; *∗*: results are statistically different (*p* < 0,05) within columns.

**Figure 5:**
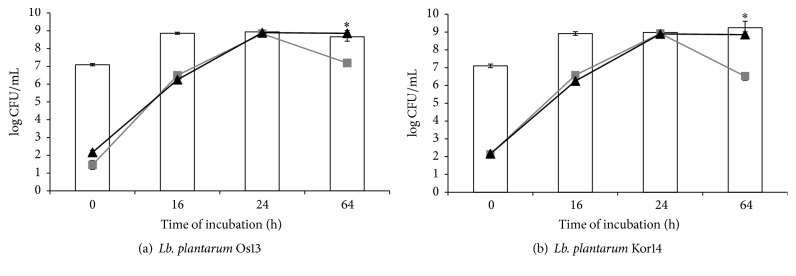
Number of the viable counts [log CFU/mL] of* E. coli* and* Lb. plantarum* Os13 and Kor14 in cocultures as follows: 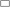: count of* Lb. plantarum* Os13 (a) and Kor14 (b); 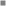:* E. coli *in coculture with* Lb. plantarum* Os13 (a) and Kor14 (b); 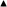:* E. coli* in control sample; *∗*: results are statistically different (*p* < 0,05) within columns.

**Figure 6:**
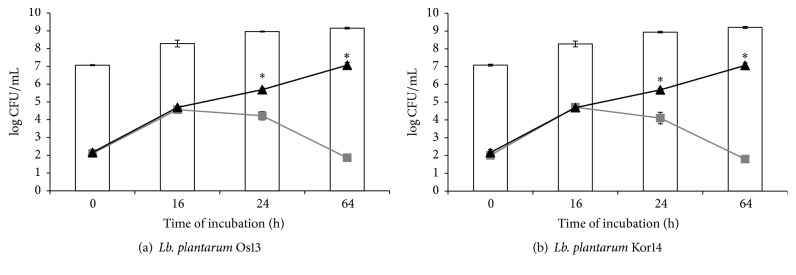
Number of the viable counts [log CFU/mL] of* S. enteritidis* and* Lb. plantarum* Os13 and Kor14 in cocultures as follows: 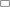: count of* Lb. plantarum* Os13 (a) and Kor14 (b); 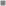:* S. enteritidis* in coculture with* Lb. plantarum* Os13 (a) and Kor14 (b); 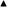:* S. enteritidis* in control sample; *∗*: results are statistically different (*p* < 0,05) within columns.

Figures  2–7 (supplementary material) show the zones of inhibition of growth of indicator microorganisms observed for all* Lb. plantarum* strain examined in the study and all indicator strains used. The antagonistic activity of the* Lb. plantarum* strains examined was diverse, which confirms that antimicrobial properties are strain-specific, as indicated by many researchers [2, 16, 19–21]. The CFN supernatants of all* Lb. plantarum* strains isolated from oscypek showed anti-*Listeria* activity, which suggests the ability of these strains to synthesize bacteriocins. This property was not observed for any* Lb. plantarum* strain isolated from korycinski cheese. The CFN supernatants of* Lb. plantarum* strains Os1, Os2, Os3, Os4, Os6, Os12, Os13, Os14, Os15, Os16, and Os17 displayed antagonistic activity also towards other indicator microorganisms, which may suggest their ability to synthesize a broad-spectrum compound. No inhibitory effect on the growth of* L. monocytogenes*,* S. enteritidis*,* B. subtilis*, and* En. faecium* was observed in the case of the CFN supernatants of strains isolated from korycinski cheese. The CFN supernatants of* Lb. plantarum* strains Kor2, Kor12, Kor13, Kor14, Kor15, Kor18, Kor19, and Kor23 showed minor antagonistic activity exclusively towards* E. coli*, and the observed zones of inhibition of the growth of this indicator microorganism had statistically significantly larger diameters than those observed in the study with* Lb. plantarum* isolated from oscypek, as shown in Figure  6 (supplementary material). Most probably the active antimicrobial substance present in the CFN supernatants of these strains has a narrow spectrum of activity.

Many researchers emphasise that the ability to synthesize antimicrobial compounds, that is, bacteriocins, is strain-specific. In the study of Sip et al. [5], only 7 out of 800 strains studied demonstrated ability to synthesize compounds inhibiting the growth of* L. monocytogenes* in a neutralized supernatant; in the study of Belicova et al. [22], none of the 125* Lactobacillus* strains showed antimicrobial activity in a neutralized supernatant [22].

Statistically significant differences between the antagonistic activity showed by strains isolated from oscypek and the antagonistic activity showed by strains isolated from korycinski cheese were noted in the study. Cluster analysis performed using Ward's agglomeration method enabled the identification of three clusters, as shown in the dendrogram (Figure 2). Clusters A and B contained all strains isolated from oscypek and the* Lb. plantarum* 299v reference strain, whereas cluster C contained strains isolated from korycinski cheese. This result may suggest that, in the case analysed, the antagonistic activity showed by* Lb. plantarum* strains is connected with the source from which a given strain was isolated.

In order to determine the number of components influencing the antagonistic activity of* Lb. plantarum* strains in the system analysed, a Principal Component Analysis was performed. Two of the components identified—PC1 and PC2—account for 80.94% and 9.23% of variability, respectively. These components defined variability within the group to the highest degree, and the loadings of these components are presented in Figure 3. PC1 correlated significantly with variables Os1–Os17 and* Lb. plantarum* 299v, whereas PC2 correlated with Kor1–Kor14. The results of the analysis indicate the existence of at least one secondary component that defines the group variability.

The antagonistic activity of LAB may be enhanced by the synthesis of antimicrobial compounds. According to Woraprayote et al. [23], the most important compounds of this type include, besides organic acids, low-molecular peptides—bacteriocins. 185 different bacteriocins were discovered before 2015, of which 17 were found in* Lb*.* plantarum *strains.* Lactococcus lactis* and* E. faecium* are the only species in which more types of bacteriocins were identified (22 in both cases). However, the majority of compounds discovered have not been classified so far, and their mechanisms of action are still investigated [23]. Plantaricins (bacteriocins identified for the first time in* Lb. plantarum*) are class IIb (two-peptide) bacteriocins. In order for a molecule to be fully effective, two peptides need to be involved, each of which individually has no antimicrobial activity or low antimicrobial activity. Researchers report that the genomes of* Lb. plantarum *strains may include sequences coding more than one bacteriocin, and each of them may be characterized by a different spectrum of activity [24–26]. Moreover, bacteriocin coding genes can be located in various structures within LAB cells—both at a locus on a bacterial chromosome and on plasmids or transposons. Genes located on plasmids can be easily transferred between cells, which facilitates the transmission of various characteristics, such as resistance to antimicrobial substances, but also the ability to synthesize such substances [27]. The spectrum of activity of plantaricins is diverse; however, most researchers consider it to be broad [7]. Hu et al. [28] report that plantaricin 163 synthesized by* Lb. plantarum* 163 inhibits the growth of both Gram-positive bacteria, that is,* S. aureus*,* L. monocytogenes*,* B. pumilus*,* B. cereus*,* Micrococcus luteus*,* Lb. thermophilus*, and* Lb. rhamnosus*, and Gram-negative bacteria—*E. coli*,* P. aeruginosa*, and* P. fluorescens *[28]. Plantaricin MG also shows antimicrobial activity towards* L. monocytogenes*,* S. aureus*,* Salmonella typhimurium*, and* E. coli *[29]. Moreover,* Lb. plantarum* capable of synthesizing bacteriocins other than plantaricin, for example, pediocin LB-B1, was also isolated from dairy products, which suggests the ability to take over elements coding bacteriocin synthesis from other cooccurring bacteria species [30]. Sip et al. [5] demonstrated that the anti-*Listeria* activity of the strains isolated from the Tatra cheese was determined by their ability to synthesize class IIa bacteriocins (the so-called pediocin-like bacteriocins), which are characterized by a broad antimicrobial spectrum and a strong inhibitory effect on the growth of* Listeria* spp. [5]. It is possible that the dependence of the antimicrobial activity of a LAB strain on the source from which it was isolated is related to the ability of these bacteria, in particular* Lb. plantarum* Os1–Os17 strains, to synthesize class IIa bacteriocins, as it is indicated by the strong anti-*Listeria* activity of these strains. The fact that strains coming from one type of regional cheese have this ability may result from the easy transfer of genes coding bacteriocin synthesis, as has been mentioned above, also between different species of bacteria.

In order to explain the observed phenomenon, it is necessary to bear in mind that bacteriocins are frequently coded by genes located in operons, and thus their synthesis is not constant [27]. Maldonado-Barragán et al. [31] reported that it is possible to induce the synthesis of plantaricin by means of cocultures with antagonistic microorganisms. Microorganisms inducing such synthesis are very diverse and include both Gram-positive and Gram-negative bacteria [31]. In the light of the above, it can be assumed that noninduced* Lb. plantarum* synthesize such biologically active compounds coded in operons as they were essential to them in the product from which they were isolated. Sip et al. [5] mentioned that* L. monocytogenes *is common in places where Tatra cheeses are produced—both on walls and floors, but also in raw milk [5]. It is possible that the* Lb. plantarum* Os1–Os17 strains examined developed strong anti-*Listeria* properties in order to effectively compete against this pathogen in the ecological niche occupied.

*Lb. plantarum* strains isolated from korycinski cheeses, in turn, showed stronger antagonistic activity against* E. coli *in comparison with* Lb. plantarum* Os1–Os17 strains, which suggests that they may have been induced by the presence of these microorganisms in the environment and thus they developed a defence mechanism against* E. coli.*

#### 3.2.2. Coculture in Skim Milk

Investigation of antimicrobial agents in the milk matrix was made to confirm the high antagonistic activity, as have been shown in well diffusion method studies. The aim of these experiments was created by the food model and examined antagonistic activity of tested strains, during bacterial optimal conditions. The tested temperature of incubation (37°) simulated the production process of fermented milk products. Figures 4, 5, and 6 show the number of indicator bacteria cells and number of* Lb. plantarum* Os13 and Kor14 cells in the tested samples.

There have been observed differences in the effectiveness of inhibiting the indicator bacteria growth depending on the species of indicator microorganism (the most effective inhibition of* L. monocytogenes* ATCC 19111 growth and the least effective* E. coli *inhibition were shown). Moreover, the differences in the antimicrobial effectiveness between strains Os13 and Kor14 were noted.

Both strains* Lb. plantarum* Os13 and Kor14 slowed down the growth of* L. monocytogenes*, although the* Lb. plantarum* Os13 is the strongest, as it was noted after 24 hours of incubation. No significant differences were observed between the number of bacteria cells of* Lb. plantarum* Os13 and Kor14 in the samples. Importantly, the conditions and the matrix used at the experiment caused a decrease in the number of* L. monocytogenes*, even in the control sample, after 24 hours of incubation and further decrease the number of culture in the following hours. However, in samples additionally inoculated with Os13 and Kor14 this process occurred more rapidly and after 64 hours of incubation, the number of* L. monocytogenes* was below the detection threshold (Figure 4). In the Østergaard et al. [32] study, it was observed that inoculation of curd cheese of both* L. monocytogenes* in the number of 2 log CFU/g and LAB culture in a quantity of 6 log CFU/g resulted in less than 4 log CFU/g population of* L. monocytogenes* after incubation, which is consistent with the results of current experiment [32].

In turn, no inhibitory effect of the Os13 and Kor14 strains on* E. coli* growth rate has been observed for the first 24 hours of incubation. Only after 64 hours of incubation, decrease in the number of* E. coli* in the samples inoculated with* Lb. plantarum*, in comparison to the control, has been noted. Moreover, it was noted that the number of* E. coli* in a sample with* Lb. plantarum *Kor14 was significantly lower than in the sample with Os13, indicating a stronger antagonism of this strain against* E. coli* (Figure 5). A rapid initial increase in the number of* E. coli* was demonstrated by Chang et al. [33], where the coculture of* E. coli* O157:H7 and* Lb. bulgaricus* in milk beverage was performed. The authors noted that, after 48 hours of incubation, the number of* E. coli* in a sample started to decrease, at different rates, depending on the LAB strain used in the study [33].

Both* Lb. plantarum* Os13 and Kor14 inhibited the growth of* S. enteritidis*, which was observed after 24 hours of incubation. There were no statistically significant differences between the numbers of* S. enteritidis*, nor between the numbers of* Lb. plantarum* in samples inoculated Os13 and Kor14. After 64 hours of incubation, the number of* S. enteritidis* was more than 5 log CFU/mL which was less than in the control (Figure 6). Other researchers have also indicated that* Lactobacillus* genus effectively inhibits growth of pathogens in milk matrix. In the study of Shady et al. [34], it was demonstrated that a cocultured LAB and indicator microorganisms, that is,* L. monocytogenes, S. typhimurium*, and* E. coli*, caused a slowdown in the growth rate of these microorganisms on average 2 log CFU/mL after 48 hours of incubation at temperature 37°C. Moreover, the results obtained in this experiment confirmed the lowest rate of growth of* L. monocytogenes* in comparison with all the indicator strains [34].

Diversified antagonism power of* Lb. plantarum* strains to the indicator exhibited in an in situ study may result from their different tolerance to the low pH environment, the presence of organic acids, hydrogen peroxide, and other antimicrobially active molecules (e.g., bacteriocins). Antimicrobial properties exhibited by* Lb. plantarum* in coculture may vary during the course of the experiment, as demonstrated by Maldonado-Barragán et al. [31]. Induction of antimicrobial peptide synthesis is possible in case of the presence in the environment of the competing organism [14]. It is worth mentioning that high anti-*Listeria* activity of both studied strains* Lb. plantarum *was observed in this experiment, which is reflected in the work of other researchers and may be related, except the potential capacity for the synthesis of bacteriocins, also with a significant pH decrease of the matrix [35]. Mariam et al. [36] also observed a slower rate of growth of indicator strain in the coculture LAB with* L. monocytogenes* in comparison with* S. enterica* subsp*. enteritidis*. It has been also shown that the number of* L. monocytogenes* cells in VBNC (viable but nonculturable) state was significantly higher than* S. enterica*. Moreover, the mean percentage of dead cells in the culture of* S. enterica* was statistically higher than number of* L. monocytogenes* dead cells throughout the incubation time [36]. This points to an additional limitation associated with the antilisterial activity of LAB, because despite effectiveness of inhibition of* L. monocytogenes* growth occurring in vitro or in situ, the cells can move to VBNC state, which is connected with their potential ability to restoration of bacterial cells.

Other authors studies indicated that LAB exhibited antagonistic activity also in relation to species of the genus* Staphylococcus *[1, 15, 34, 37, 38]. The effectiveness of growth inhibition, observed by researchers both in vitro and in situ, varies from very weak to very strong. Results are dependent on strains and measurement methods applied. Researchers observed the differentiation of antagonism between both LAB and* S. aureus* strains [1, 37]. Notably,* L. plantarum* are proven potential LAB candidates having antagonistic activity towards* S. aureus* [39, 40]. Although, in our study, we did not investigate the antagonistic activity towards* S. aureus*, considering the high frequency of milk products infections with staphylococci, further studies should be focused on that topic.

## 4. Conclusions

The results of this in vitro study showed that all* Lb. plantarum* strains isolated from cheese samples were active against indicator strains, also against potential pathogens. Moreover, each strain from oscypek produced broad spectrum, and a few strains isolated from korycinski cheese produced a narrow spectrum of antimicrobial agents, other than organic acids and hydrogen peroxide. Antimicrobial properties were strain-related. These results led us to further characterize and purify the antimicrobial compound produced by the studied strains, in purpose to characterize the nature of them. Since all* Lb. plantarum* strains showed strong antimicrobial activities against a wide range of potential pathogens, especially* L. monocytogenes*, they could be considered as good candidates for protective cultures to extend durability of fermented foods.

## Supplementary Material

Supplementary Material contain the diameters of measured inhibition growth zones observed in tests with various indicator strains and with various form of Lb. plantarum strains.

## References

[1] Arena M. P., Silvain A., Normanno G. (2016). Use of *Lactobacillus plantarum* strains as a bio-control strategy against food-borne pathogenic microorganisms. *Frontiers in Microbiology*.

[2] Cortés-Zavaleta O., López-Malo A., Hernández-Mendoza A., García H. S. (2014). Antifungal activity of lactobacilli and its relationship with 3-phenyllactic acid production. *International Journal of Food Microbiology*.

[3] Neal-McKinney J. M., Lu X., Duong T. (2012). Production of organic acids by probiotic lactobacilli can be used to reduce pathogen load in poultry. *PLoS ONE*.

[4] Rodríguez-Pazo N., Vázquez-Araújo L., Pérez-Rodríguez N., Cortés-Diéguez S., Domínguez J. M. (2013). Cell-free supernatants obtained from fermentation of cheese whey hydrolyzates and phenylpyruvic acid by *Lactobacillus plantarum* as a source of antimicrobial compounds, bacteriocins, and natural aromas. *Applied Biochemistry and Biotechnology*.

[5] Sip A., Więckowicz M., Olejnik-Schmidt A., Grajek W. (2012). Anti-Listeria activity of lactic acid bacteria isolated from golka, a regional cheese produced in Poland. *Food Control*.

[6] Altay F., Karbancıoglu-Güler F., Daskaya-Dikmen C., Heperkan D. (2013). A review on traditional turkish fermented non-alcoholic beverages: microbiota, fermentation process and quality characteristics. *International Journal of Food Microbiology*.

[7] da Silva Sabo S., Vitolo M., González J. M. D., de Souza Oliveira R. P. (2014). Overview of *Lactobacillus plantarum* as a promising bacteriocin producer among lactic acid bacteria. *Food Research International*.

[8] Guidone A., Zotta T., Ross R. P. (2014). Functional properties of *Lactobacillus plantarum* strains: a multivariate screening study. *LWT—Food Science and Technology*.

[9] Rantsiou K., Urso R., Dolci P., Comi G., Cocolin L. (2008). Microflora of feta cheese from four Greek manufacturers. *International Journal of Food Microbiology*.

[10] Reis J. A., Paula A. T., Casarotti S. N., Penna A. L. B. (2012). Lactic acid bacteria antimicrobial compounds: characteristics and applications. *Food Engineering Reviews*.

[11] Brooks J. C., Martinez B., Stratton J., Bianchini A., Krokstrom R., Hutkins R. (2012). Survey of raw milk cheeses for microbiological quality and prevalence of foodborne pathogens. *Food Microbiology*.

[12] Hartmann H. A., Wilke T., Erdmann R. (2011). Efficacy of bacteriocin-containing cell-free culture supernatants from lactic acid bacteria to control *Listeria monocytogenes* in food. *International Journal of Food Microbiology*.

[13] Berta G., Chebeňová V., Brežná B., Pangallo D., Valík L., Kuchta T. (2009). Identification of lactic acid bacteria in Slovakian bryndza cheese. *Journal of Food and Nutrition Research*.

[14] Ghahremani E., Mardani M., Rezapour S. (2015). Phenotypic and genotypic characterization of lactic acid bacteria from traditional cheese in khorramabad city of iran with probiotic potential. *Applied Biochemistry and Biotechnology*.

[15] Mariam S. H., Zegeye N., Tariku T., Andargie E., Endalafer N., Aseffa A. (2014). Potential of cell-free supernatants from cultures of selected lactic acid bacteria and yeast obtained from local fermented foods as inhibitors of *Listeria monocytogenes*, *Salmonella spp.* and *Staphylococcus aureus*. *BMC Research Notes*.

[16] Složilová I., Purkrtová S., Kosová M., Mihulová M., Šviráková E., Demnerová K. (2014). Antilisterial activity of lactic acid bacteria against *Listeria monocytogenes* strains originating from different sources. *Czech Journal of Food Sciences*.

[17] Li P., Gu Q., Zhou Q. (2016). Complete genome sequence of *Lactobacillus plantarum* LZ206, a potential probiotic strain with antimicrobial activity against food-borne pathogenic microorganisms. *Journal of Biotechnology*.

[18] Mufandaedza J., Viljoen B. C., Feresu S. B., Gadaga T. H. (2006). Antimicrobial properties of lactic acid bacteria and yeast-LAB cultures isolated from traditional fermented milk against pathogenic *Escherichia coli* and *Salmonella enteritidis* strains. *International Journal of Food Microbiology*.

[19] Yang E., Fan L., Jiang Y., Doucette C., Fillmore S. (2012). Antimicrobial activity of bacteriocin-producing lactic acid bacteria isolated from cheeses and yogurts. *AMB Express*.

[20] Ünlü G., Nielsen B., Ionita C. (2016). Inhibition of *Listeria monocytogenes* in hot dogs by surface application of freeze-dried bacteriocin-containing powders from lactic acid bacteria. *Probiotics and Antimicrobial Proteins*.

[21] Pisano M. B., Patrignani F., Cosentino S., Guerzoni M. E., Franz C. M. A. P., Holzapfel W. H. (2011). Diversity and functional properties of *Lactobacillus plantarum*-group strains isolated from Italian cheese products. *Dairy Science and Technology*.

[22] Belicová A., Mikulášová M., Dušinský R. (2013). Probiotic potential and safety properties of *Lactobacillus plantarum* from Slovak Bryndza cheese. *BioMed Research International*.

[23] Woraprayote W., Malila Y., Sorapukdee S., Swetwiwathana A., Benjakul S., Visessanguan W. (2016). Bacteriocins from lactic acid bacteria and their applications in meat and meat products. *Meat Science*.

[24] Diep D. B., Straume D., Kjos M., Torres C., Nes I. F. (2009). An overview of the mosaic bacteriocin pln loci from *Lactobacillus plantarum*. *Peptides*.

[25] Rogne P., Haugen C., Fimland G., Nissen-Meyer J., Kristiansen P. E. (2009). Three-dimensional structure of the two-peptide bacteriocin plantaricin JK. *Peptides*.

[26] Ehrmann M. A., Remiger A., Eijsink V. G. H., Vogel R. F. (2000). A gene cluster encoding plantaricin 1.25*β* and other bacteriocin-like peptides in *Lactobacillus plantarum* TMW1.25. *Biochimica et Biophysica Acta—Gene Structure and Expression*.

[27] Todorov S. D. (2009). Bacteriocins from *Lactobacillus plantarum*—production, genetic organization and mode of action. *Brazilian Journal of Microbiology*.

[28] Hu M., Zhao H., Zhang C., Yu J., Lu Z. (2013). Purification and characterization of plantaricin 163, a novel bacteriocin produced by *Lactobacillus plantarum* 163 isolated from traditional Chinese fermented vegetables. *Journal of Agricultural and Food Chemistry*.

[29] Gong H. S., Meng X. C., Wang H. (2010). Plantaricin MG active against gram-negative bacteria produced by *Lactobacillus plantarum* KLDS1.0391 isolated from 'Jiaoke', a traditional fermented cream from China. *Food Control*.

[30] Xie Y., An H., Hao Y. (2011). Characterization of an anti-Listeria bacteriocin produced by *Lactobacillus plantarum* LB-B1 isolated from koumiss, a traditionally fermented dairy product from China. *Food Control*.

[31] Maldonado-Barragán A., Caballero-Guerrero B., Lucena-Padrós H., Ruiz-Barba J. L. (2013). Induction of bacteriocin production by coculture is widespread among plantaricin-producing *Lactobacillus plantarum* strains with different regulatory operons. *Food Microbiology*.

[32] Østergaard N. B., Eklöw A., Dalgaard P. (2014). Modelling the effect of lactic acid bacteria from starter- and aroma culture on growth of *Listeria monocytogenes* in cottage cheese. *International Journal of Food Microbiology*.

[33] Chang J.-H., Shim Y. Y., Cha S.-K., Chee K. M. (2010). Probiotic characteristics of lactic acid bacteria isolated from kimchi. *Journal of Applied Microbiology*.

[34] Shady T. S. M., Eman H. A., El-Badrawy E. E. Y. (1999). The inhibitory effect of lactic starter culture against food borne pathogenic bacteria in skim milk. *Pakistan Journal of Biological Sciences*.

[35] Saá Ibusquiza P., Nierop Groot M., Debán-Valles A., Abee T., den Besten H. M. W. (2015). Impact of growth conditions and role of sigB on *Listeria monocytogenes* fitness in single and mixed biofilms cultured with *Lactobacillus plantarum*. *Food Research International*.

[36] Mariam S. H., Zegeye N., Aseffa A., Howe R. (2017). Diffusible substances from lactic acid bacterial cultures exert strong inhibitory effects on *Listeria monocytogenes* and *Salmonella enterica* serovar *enteritidi*s in a co-culture model. *BMC Microbiology*.

[37] Chaalel A., Riazi A., Dubois-Dauphin R., Thonart P. (2015). Screening of plantaricin EF and JK in an Algerian *Lactobacillus plantarum* isolate. *Asian Pacific Journal of Tropical Disease*.

[38] Datta S., Namal K. S., Paras P., Sharma P., Shaikh N., Nagar J. (2013). Antagonistic activity of lactic acid bacteria from dairy products. *International Journal of Pure & Applied Bioscience*.

[39] Shah N., Patel A., Ambalam P., Holst O., Ljungh A., Prajapati J. (2016). Determination of an antimicrobial activity of Weissella confusa, *Lactobacillus fermentum*, and *Lactobacillus plantarum* against clinical pathogenic strains of *Escherichia coli* and *Staphylococcus aureus* in co-culture. *Annals of Microbiology*.

[40] Nawaz S. K., Riaz S., Hasnain S. (2009). Screening for anti-methicillin resistant *Staphylococcus aureus* (MRSA) bacteriocin producing bacteria. *African Journal of Biotechnology*.

