# Supplementary material for: Comparison of Antibacterial Activity of Lactobacillus plantarum Strains Isolated from Two Different Kinds of Regional Cheeses from Poland: Oscypek and Korycinski Cheese
Source: Biomed Res Int. 2017 May 24;2017:6820369. doi: 10.1155/2017/6820369 (PMC5463104; doi:10.1155/2017/6820369)
Supplement: Supplementary file 1 — Supplementary Material contain the diameters of measured inhibition growth zones observed in tests with various indicator strains and with various form of Lb. plantarum strains. [file 6820369.f1.docx]

**Supplementary material**

Fig. 1. The average diameter of measured inhibition growth zone observed in testing with various indicator strains and with various form of *Lb. plantarum* strains; L.m. 15313 –*Listeria monocytogenes* ATCC 15313, L.m. 7644 – *Listeria monocytogenes* ATCC 7644, L.m. 19111 - *Listeria monocytogenes* ATCC 19111, S. ent. 13076 *– Salmonella enteritidis* ATCC 13076; form of *Lb. plantarum* strain used: WBC – whole bacteria culture, CFS – cell-free supernatant, CFN – cell-free neutralized, catalase treated supernatant

Fig. 2. The average zones of inhibition of *L. monocytogenes* ATCC 15313 by *Lb. plantarum* strains; form of *Lb. plantarum* strain used: WBC – whole bacteria culture, CFS – cell-free supernatant, CFN – cell-free neutralized, catalase treated supernatant

Fig. 3. The average zones of inhibition growth of *L. monocytogenes* ATCC 7644 by *Lb. plantarum* strains; form of *Lb. plantarum* strain used: WBC – whole bacteria culture, CFS – cell-free supernatant, CFN – cell-free neutralized, catalase treated supernatant

Fig. 4. The average zones of inhibition growth of *L. monocytogenes* ATCC 19111 by *Lb. plantarum* strains; form of *Lb. plantarum* strain used: WBC – whole bacteria culture, CFS – cell-free supernatant, CFN – cell-free neutralized, catalase treated supernatant

Fig. 5. The average zone of inhibition growth zone of *B. subtilis* by *Lb. plantarum* strains; form of *Lb. plantarum* strain used: WBC – whole bacteria culture, CFS – cell-free supernatant, CFN – cell-free neutralized, catalase treated supernatant

Fig. 6. The average zones of inhibition growth of *E. coli* by *Lb. plantarum* strains; form of *Lb. plantarum* strain used: WBC – whole bacteria culture, CFS – cell-free supernatant, CFN – cell-free neutralized, catalase treated supernatant

Fig. 7. The average zones of inhibition growth of *En. faecium* by *Lb. plantarum* strains; form of *Lb. plantarum* strain used: WBC – whole bacteria culture, CFS – cell-free supernatant, CFN – cell-free neutralized, catalase treated supernatant

Fig. 8. The average zones of inhibition growth of *S. enteritidis* ATCC 13076 by *Lb. plantarum* strains; form of *Lb. plantarum* strain used: WBC – whole bacteria culture, CFS – cell-free supernatant, CFN – cell-free neutralized, catalase treated supernatant
